# Intermittent dysphagia revealing a lateropharyngeal neurofibroma in a child: Case report: A case report

**DOI:** 10.1016/j.amsu.2021.102438

**Published:** 2021-06-01

**Authors:** Wydadi Omar, Ahmed Brahim Ahmedou, Oukessou Youssef, Rouadi Sami, Redallah Abada, Roubal Mohamed, Mahtar Mohamed

**Affiliations:** ENT, Head and Neck Surgery Department, Ibn Rochd UniversityHospital, Faculty of Medicine and Pharmacy, Hassan II, Casablanca, Morocco

**Keywords:** Dysphaia, Type 1 neurofibromatosis, Neck region, Child, Abstention therapeutic

## Abstract

**Introduction:**

Neurofibromatosis type 1 (NF1) is an disorder characterised by various phenotypic features like hyperpigmented spots, neurofibromas, Lisch nodules, skeletal abnormalities and tendency to develop neoplasms.

**Case presentation:**

We present the case of a 12-year-old patient referred by his pediatrician for intermittent dysphagia and a sensation of food attachment, in whom several café-au-lait spots on the body had been found, and a case of type 1 neurofibromatosis in the patient's siblings. The decision was to closely follow-up the patient, the progression of his symptoms and the size of the cervical neurofibroma. The patient's current follow-up has been two years, with a minimal increase in the frequency of episodes of dysphagia, and with Ct-scan performed every year. No major growth of the cervical mass was noted.

**Discussion:**

Neurofibromatosis type 1 (NF1) is an autosomal dominant disorder characterised by various phenotypic features like hyperpigmented spots, neurofibromas, Lisch nodules, skeletal abnormalities and tendency to develop neoplasms.

**Conclusion:**

The treatment is not codified and abstention therapeutic may be a wise decision.

## Highlights

• NF1 shows an autosomal dominant pattern of inheritance and wide phenotypical variability.
• Neurofibromatosis type 1 is a neurogenetic disorder.
• The cervical soft tissues may also be the primary site for tumors.
• The treatment is not codified.
• Abstention therapeutic may be a wise decision.

## 1 Introduction

We present a case in accordance with SCARE 2020 criteria [1]. Neurofibromatosis (von Recklinghausen disease) was first described as a distinct entity by von Recklinghausen in 1882 [2].

Neurofibromatosis type 1 is a neurogenetic disorder with a birth prevalence estimated around 1:2500 [3]. While it is known that incidence of head and neck tumors in NF1 is approximately 25 ± 30% the incidence of cervical soft tissue neoplasms in children is not known [3]. The cervical soft tissues may also be the primary site for tumors. NF1 shows an autosomal dominant pattern of inheritance and wide phenotypical variability. Café-au-lait spots (CALs), cutaneous and/or subcutaneous neurofibromas (CNFs/SCNFs), skin fold freckling, skeletal abnormalities and Lisch nodules of the iris are its main clinical features. NF1 patients have an increased risk of learning and intellectual disabilities as well as tumors of the nervous system and other organs [4].

Neurofibromatosis type 1 (NF1) is an disorder characterised by various phenotypic features like hyperpigmented spots, neurofibromas, Lisch nodules, skeletal abnormalities and tendency to develop neoplasms [5].

The condition is characterised by multiple skin lesions such as café-au-lait macules and neurofibromas growing along the parent nerves.

Plexiform neurofibromas represent a special variant of NF-1 in which neurofibromas can arise from multiple nerves as bulging and deforming masses [6]. The treatment is a therapeutic challenge.

### 1.1 Case report

**Fig. 1:**
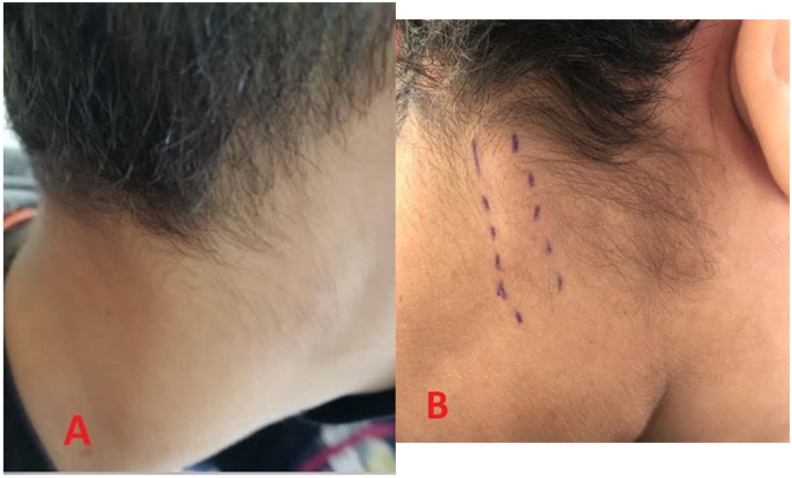
A: Cervical swelling located in the right part of the neck, under the sternocleid, B: indurated cord in the posterior part of the neck.

**Fig. 2:**
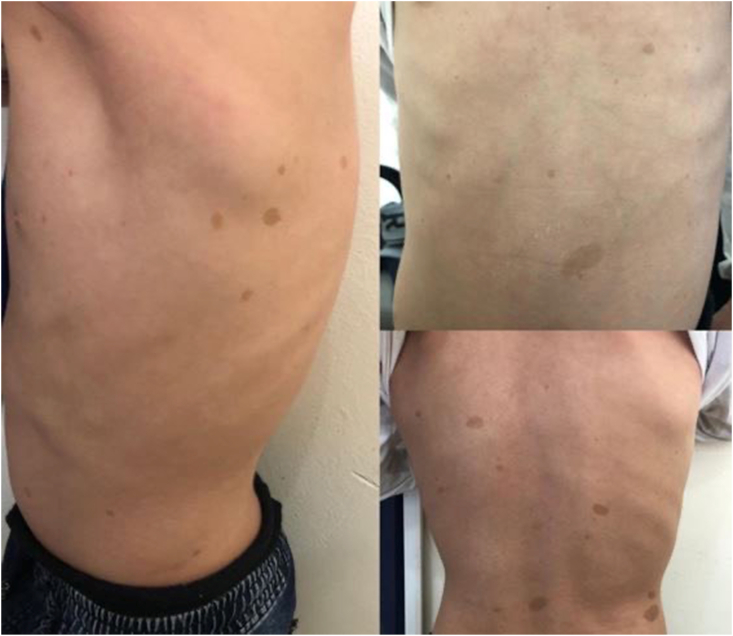
Café au lait spots in the upper body of the patient.

**Fig. 3:**
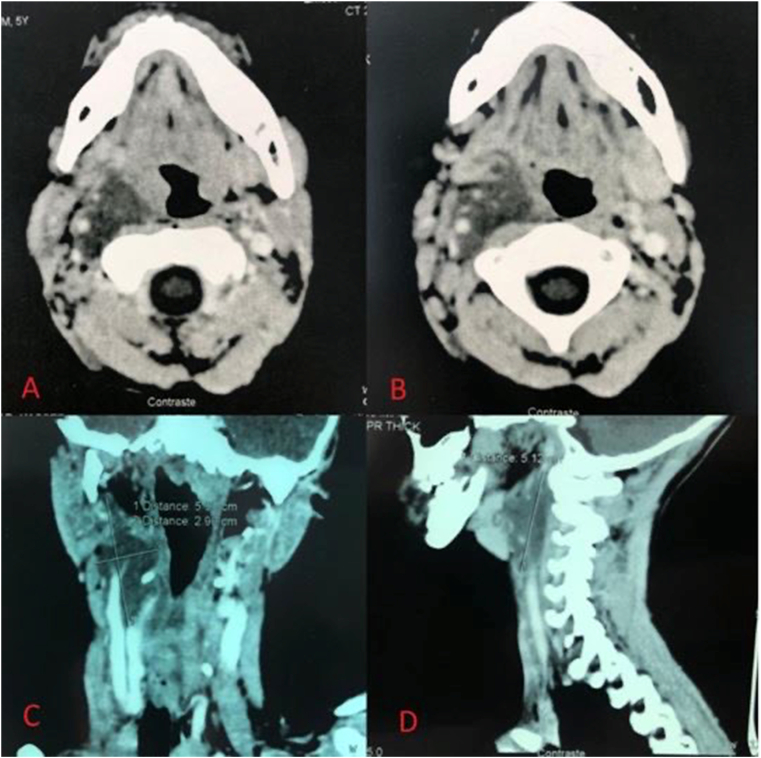
CT-scan showing an irregular hypodense mass, laterally to the -larynx, crossed by the jugulo-carotid vessels. **A-B**: Axial cut. C-coronal. D-Sagital cut.

We present the case of a 12-year-old patient admitted to our department for Intermittent dysphagia and a sensation of food attachment, in whom several café-au-lait spots on the body had been found, and a case of type 1 neurofibromatosis in the patient's siblings. Our general examination had to be complete, and was oriented towards this neurofibromatosis given the clinical information. The examination found a right cervical swelling in the middle part of the sternocleidomastoid muscle, slowly evolving according to the parents (Fig. 1 A), and an indurated painful cord on the course of the posterior auricular nerve (Fig. 1 B). Oropharyngeal examination and nasofibroscopy were poor in clinical information. On the thorax, abdomen and back of the patient, the presence of eight café au lait spots larger than 5 mm and several others of small size were noted (Fig. 2). The cerebral and cervical CT showed the presence of a right retro mastoid subcutaneous mass of 40.7 mm; and a lateral pharyngolaryngeal tissue process of 59.6 mm, medial to the parotid crossed by the jugulo-carotid vessels (Fig. 3).

At this point, several criteria were in favor of type 1 neurofibromatosis, namely: a history of this disease in the siblings, Eight café-au-lait spots greater than 5 mm on the body (Fig. 2), a nodular retro-mastoid neurofibroma (Fig. 1 B), removed surgically and confirmed by histologie, a strong suspicion of plexiform neurofibroma (the laryngopharyngeal mass) (Fig. 3), which constituted the therapeutic challenge of the joung patient. A paraclinical assessment searching for gravity signs was made: dosage of urinary cathecolamines, which was normal eliminating the pheochromocytoma; a normal blood pressure and an Ophthalmological examination without particularities. An x-ray of the tibia/fibula bones was done without abnormalities.

Neither biopsy nor surgical procedure for this probable cervical neurofibroma were retained in a multidisciplinary meeting, in view of his cervical location, hard to reach by external or endolaryngeal approach, the surgical risks on the jugulo-carotid vessels, comorbidities or sequelae that would result from surgery. The decision was to closely follow-up the patient, the progression of his symptoms and the size of the cervical neurofibroma. The endolaryngeal biopsy was planned if the mass grew and arrived closely to the endolaryngeal or endopharyngeal mucosa. The patient's current follow-up has been two years, with a minimal increase in the frequency of episodes of dysphagia, and with Ct-scan performed every year. No major growth of the cervical mass was noted.

## 2 Discussion

Neurofibromatosis (von Recklinghausen disease) was first described as a distinct entity by von Recklinghausen in 1882 [2].

Especially in paediatric and adolescence population not more than 2% develop clinical symptoms. The majority of these tumors is found in the parapharyngeal, carotid, or retropharyngeal spaces. All described cervical soft tissue tumors are plexiform neurofibromas, but the appearances of these tumors vary. NF1 gene maps on chromosome 17q11.2. Microdeletions of this region are responsible for NF1 microdeletion syndrome, observed in 4.2% of all NF1 patients. Large deletions of NF1 and its flanking regions have been associated with more severe phenotype than NF1 general population. Four types (1, 2, 3 and atypical) of such large NF1 deletions have been reported so far, with differences in size, breakpoint location, number of genes deleted and somatic mosaicism [7].

Type-1 is the most frequent (70–80%), while 8–10% are atypical [8].

The tumor is most often supraglottic, rarely infraglottic. Sarcomatous degeneration of the cervical neurofibromas or primary malignant tumors of the cervical soft tissues are rare, especially in children, although reported [9,10].

The diagnosis of NF1 requires two or more of the following [11]:

1. Six or more cafe au lait macules over 5 mm in greatest diameter in prepubertal individuals and over 15 mm in greatest diameter in postpubertal individuals
2. Two or more neurofibromas of any type or one plexiform neurofibroma
3. Freckling in the axillary or inguinal regions
4. Optic glioma
5. Two or more Lisch nodules (iris hamartomas)
6. A distinctive osseous lesion such as sphenoid dysplasia or thinning of long bone cortex, with or without pseudarthrosis
7. A first-degree relative (parent, sibling, or offspring) with NF1 by the above criteria.

The clinical presentation of these entities (pharyngeal localisation) is usually not related to the tumor size. Dysphagia, vocal cord paralysis, hoarseness of voice, and heart rate changes are the most common manifestations.

Preoperative diagnosis of a neurofibroma arising from cervical vagus nerve is challenging. The differential diagnosis includes enlarged lymph node and other neurogenic tumors [4]. The diff erential diagnosis of vagal nerve neurofi bromas, other nevre sheath tumors, includes lipomas, branchial cleft cysts, thyroid adeno-mas, benign or malignant lymphadenopathy, sarcomas, salivary gland tumors, metastatic carcinomas, abscesses, carotid body tumors, and tuberculous lymphadenitis [12]. The initial evaluation of a patient with a suspected cervical nerve neurofi broma should include history assessment, focusing on stigmata or a family history of neurocutaneous disease [13,14]. Ultrasonography may also be helpful in establishing whether the tumor lies between the jugular vein and the carotid artery [13]. Ultrasonography-or computed tomography-guided biopsy may indicate spindle cells, suggesting a nerve sheath tumor. These mass can show in CT and MR studies as well-differentiated fibromas, or as an almost uniform mass of the involved organs [15]. Biondetti et al. [16] and Bass et al. [17] observed the symmetric homogeneous appearance of neurofibromas. Due to their anatomical relationship to the neural structures and major blood vessels, operating neurofibromas with parapharyngeal extension is a surgical challenge.

Microscopically, neurofi bromas display well-diff erentiated, fusiform, spindle cells, without frequent mitoses. Neurofibromas exhibit a typical homogeneous appearance, with a loose arrangement of spindle-shaped cells in a clear mucinous matrix (Antoni B-type tissue) [18]. The current lack of literature on FSNF1 and Non-FSNF1 involving is responsible for the limited knowledge in deciding the most favourable treatment plan and results.

## 3 Conclusion

It is up to the surgeon to have a global vision and to evaluate the risk-benefit balance before proposing to propose a surgery. The evolution of the lesions during puberty and into adulthood should be taken into account when discussing the best time to perform surgery. In our case, therapeutic abstention and surveillance was the decision with a stable disease for 2 years.

## Conflicts of interest

The authors declare having no conflicts of interest for this article.

## Funding

None.

## Ethical approval

I declare on my honor that the ethical approval has been exempted by my establishment.

## Consent

Written informed consent for publication of their clinical details and/or clinical images was obtained from the patient's parents.

## Author contribution

Wydadi Omar: Corresponding author writing the paper, Ahmed Brahim Ahmedou: writing the paper, Youssef Oukessou: study concept, Sami Rouadi: study concept, Redallah Abada: study concept, Mohamed Roubal: correction of the paper, Mohamed Mahtar: correction of the paper, Registration of Research Studies.

## Registration of Research Studies

Researchregistry5198.

## Guarantor

The Guarantor is the one or more people who accept full responsibility for the work and/or the conduct of the study, had access to the data, and controlled the decision to publish.
